# Comparison of the similarity between two quantum images

**DOI:** 10.1038/s41598-022-11863-9

**Published:** 2022-05-11

**Authors:** You-hang Liu, Zai-dong Qi, Qiang Liu

**Affiliations:** Quantum Computing Research Department, Inspur Academy of Science and Technology, Jinan, 250014 China

**Keywords:** Quantum information, Qubits, Quantum simulation

## Abstract

Comparing the similarity between digital images is an important subroutine in various image processing algorithms. In this study, we present three quantum algorithms for comparing the similarity between two quantum images. These algorithms are applied to binary, grey and color images for the first time. Without considering the image preparation, the proposed algorithms achieve exponential acceleration than the existing quantum and classical methods in all three cases. At the end of this paper, an experiment based on the real quantum computer of IBMQ and simulations verify the effectiveness of the algorithms.

## Introduction

After Google realizing “quantum supremacy” in 2019^1^, quantum computing has aroused the great interest of the public. Exploiting quantum mechanics such as superposition and entanglement, quantum computing shows an overwhelming computing power advantage in some certain problems. Nonetheless, on account of the few quantum algorithms, these problems were extremely limited for more than a decade after the concept of the quantum computer was first proposed by Richard Feynman in 1982^2^. Nowadays, after decades of development, both quantum computer hardware and architecture have got some revolutionary breakthrough^3–6^, and quantum computing has entered a new era called “Noisy Intermediate-Scale Quantum (NISQ)”^7^. Researches on quantum algorithms also obtained some remarkable results^8–12^. Thanks to increasing demand for efficient image processing algorithms, quantum image processing (QImP) is one of the hot topics.

For QImP, the first issue to be addressed is how to represent an image in a quantum computer. In recent years, a variety of quantum image representation (QImR) methods have been developed, such as quantum lattice^13^, entangled image^14^, flexible representation of quantum images (FRQI)^15^, a novel enhanced quantum representation of digital images (NEQR)^16^ and their variants^17,18^. The former two QImR methods were proposed in an early stage and did not benefit from quantum speed-up^19^. Compared with FRQI, NEQR and its variants have advantages in both image preparation and color processing^16,20^, which is vital in today’s computer vision technology.

Based on different QImR methods, various QImP algorithms were proposed to solve problems in different fields of image processing, e.g. edge detection^19,21^, image watermarking^22–25^, image scaling^26,27^, and etc. As for assessing the similarity between two quantum images, most researches were based on FRQI and its variants^28–31^. There was a research comparing images based on NEQR and its variant, a novel quantum representation of color digital images (NCQI), by using quantum amplitude amplifier and estimation in 2019^20^. Nevertheless, the main component of quantum amplitude amplifier, an oracle which only flips the amplitude sign of the searched input, still needs to be given as a black box to the algorithm^32,33^. In addition, the binarization of grey and color images in^20^ is slightly deficient due to the lack of available quantum circuits. In this paper, we present three novel algorithms on comparing the similarity between two quantum images based on NEQR and its variants. Our algorithms consist entirely of general quantum gate set, benefit from additional quantum accelerations, and are readily applicable on binary, grey, and color quantum images.

In the rest of this paper, we briefly described the representation methods based on NEQR and NCQI in “NEQR and NCQI” section. The proposed algorithms were presented in “Compare the similarity between two quantum images” section. In “Algorithm complexity” section, we analyzed the time complexity of our algorithms and gave a comparison with classical image processing and existing quantum methods. Finally, several experiment results were given in “Calculation results” section to illustrate the validity of the algorithms.

## NEQR and NCQI

In this research, the representation of binary and grey images is based on NEQR, and the representation of color images is based on NCQI.

### NEQR

Supposing an image is composed of ${2}^{n}\times {2}^{n}$ pixels and has a grey range of ${2}^{q}$, it can be represented as^16^:

1 $$|{\mathrm{Image}}_{grey}> =\frac{1}{{2}^{n}}\sum_{Y=0}^{{2}^{n}-1}\sum_{X=0}^{{2}^{n}-1}\begin{array}{c}q-1\\ \otimes \\ i=0\end{array}|{C}_{YX}^{i}>|YX>$$

$|{C}_{YX}>$ encodes the grey-scale value and $|YX>$ encodes the pixel positions. The length of $|{C}_{YX}>$ is q for representing a grey range of ${2}^{q}$. Binary images can be regarded as a special case taking q = 1. After converting to binary format, the length of $|YX>$ is 2n.

### NCQI

Supposing a RGB color image is composed of ${2}^{n}\times {2}^{n}$ pixels and every RGB component has a range of ${2}^{q}$, then it can be represented as^17^:

2 $$|{\mathrm{Image}}_{color}>=\frac{1}{{2}^{n}}\sum_{Y=0}^{{2}^{n}-1}\sum_{X=0}^{{2}^{n}-1}\begin{array}{c}q-1\\ \otimes \\ i=0\end{array}|{R}_{YX}^{i}{G}_{YX}^{i}{B}_{YX}^{i}>|YX>$$

$|YX>$ encodes the pixel positions and $|{R}_{YX}>$, $|{G}_{YX}>$, $|{B}_{YX}>$ encodes the red-scale, green-scale, and blue-scale value respectively. The length of $|{R}_{YX}{G}_{YX}{B}_{YX}>$ is 3q, indicating that the range of red, green and blue is ${2}^{q}$ respectively. After converting to binary format, the length of $|YX>$ is 2n.

## Compare the similarity between two quantum images

**Figure 1:**
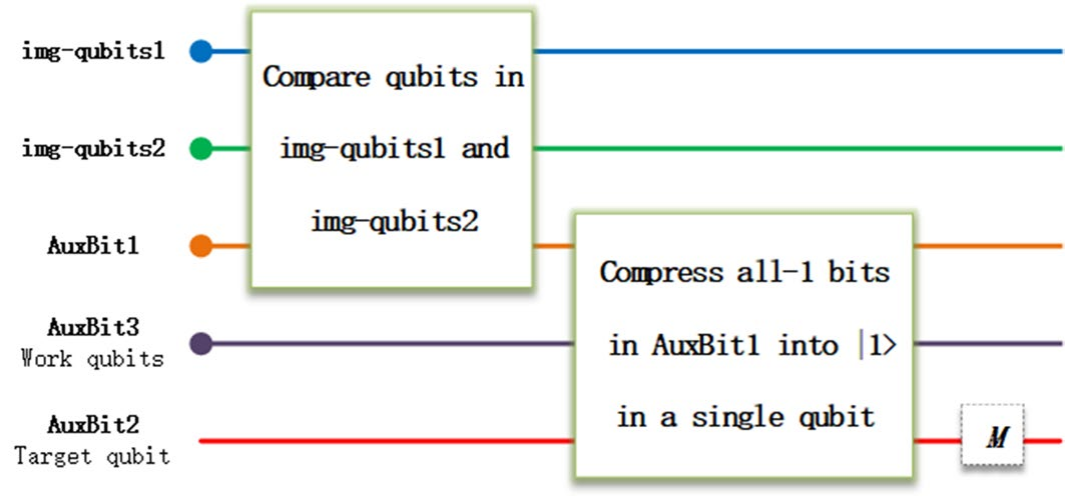
Three steps in comparing the similarity between two quantum images. Different colors represent qubits carrying different functions. The dot on endpoint indicates a multi qubits string. M represents measurement operation.

After preparing two quantum images into qubits 1 (img-qubits1) and qubits 2 (img-qubits2) based on NEQR or NCQI, similarity assessment between these two images is summarized into three steps, as shown in Fig. 1. In the following, we present the detailed information for the applications on binary, grey, and color images.

### Binary images

#### **Algorithm 1**

Compare the similarity between two binary images.

Step 1: Compare all qubits in img-qubits1 and img-qubits2 one by one and generate auxiliary qubits 1 (AuxBit1);
Step 2: Compress all-1 qubit strings $|11\dots 11>$ of AuxBit1 into $|1>$ of a single qubit (AuxBit2);
Step 3: Measure AuxBit2 and calculate the similarity s with $\mathrm{s}=\mathrm{p}\times {2}^{2n}$, where p is the probability of getting $|1>$ and ${2}^{2n}$ is the size of images.

Binary images have only two pixel values, namely 0 and 1, which could be regarded as a special case of NEQR. Thus, when comparing two pixels, there are only two results, totally the same and completely different.

**Figure 2:**
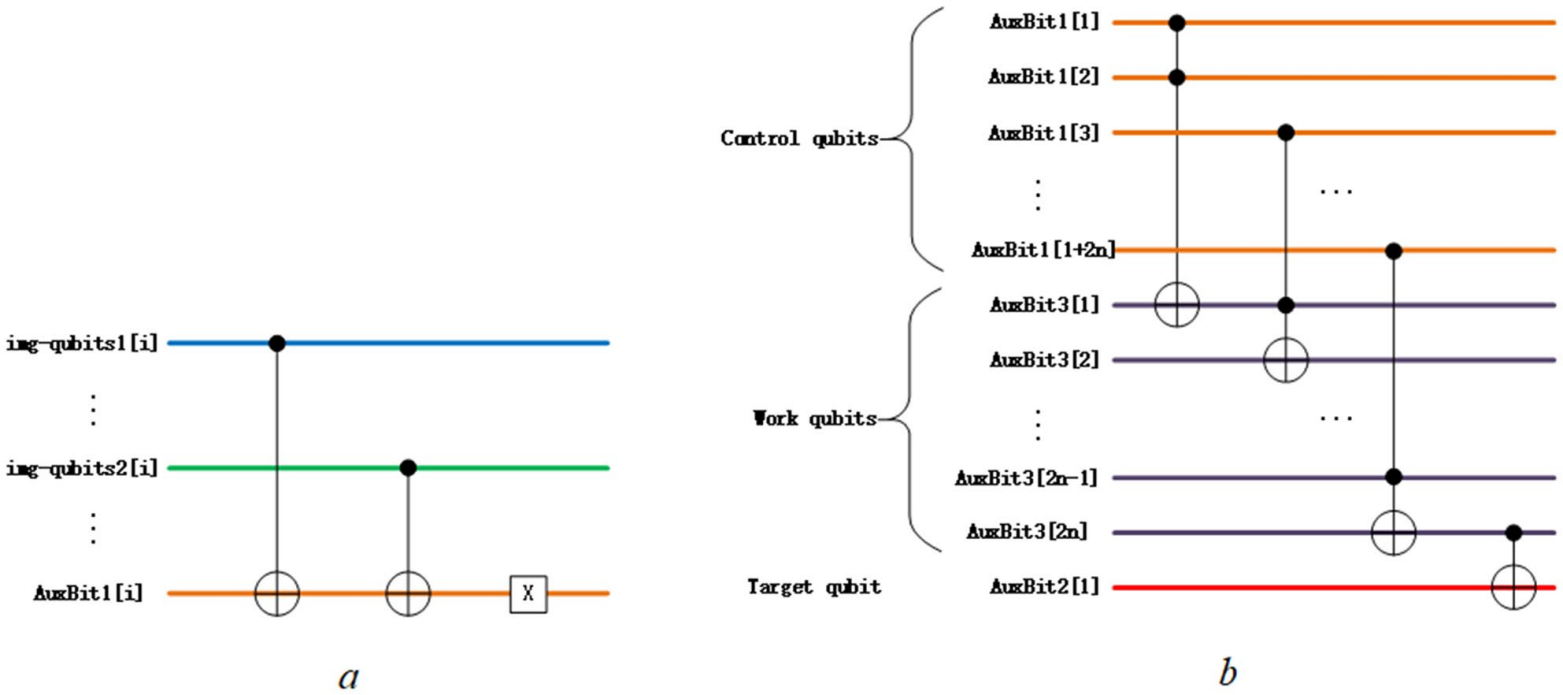
(**a**) The quantum circuit for comparing qubits from img-qubits1 and img-qubits2. This circuit defines the mapping between two NEQR image strings and AuxBit1. The output is XNOR of two input. (**b**) The quantum circuit that compresses |11…11 > to |1 > and the other states to |0 > . This circuit equals the Control-Not gate conditioning on the whole AuxBit1. The qubits with the same color as in Fig. 1 have the same function.

As pixel values are represented using binary qubits in NEQR, it’s intuitive to think of comparing the pixel value qubits and the position qubits simultaneously. Only candidates having totally identical qubit strings have a contribution to the final result. The step 1 is to compare all qubits in img-qubits1 and img-qubits2 one by one and generate auxiliary qubits 1 (AuxBit1). Focusing on comparison between two qubits (one from imag-qubits1, and the other from the same site of imag-qubits2), if qubits for comparing are identical, the auxiliary qubit is set to 1; if different, the auxiliary qubit is set to 0, as shown in the truth table (Table 1). This operation is realized with two Control-Not (C-Not) gates and one NOT (X) gate, as shown in Fig. 2a.

**Table 1:** Truth table for comparing two qubits.

img-qubits1[i]	img-qubits2[i]	AuxBit1[i]
0	0	1
0	1	0
1	0	0
1	1	1

The value of AuxBit1[i] would be set to 1 only if the values of img-qubits1[i] and img-qubits2[i] are the same.

It can be proved that the probability of getting all-1 qubit string (the following is written as $|11\dots 11>$ for simplification) in measuring Auxbit1 equals $\frac{s}{{2}^{2n}}$, where ${2}^{2n}$ is the total number of pixels in a single image. Please refer to supplementary information for the detailed process. However, because of the ineluctable errors included in a real quantum computer, there may exist ${2}^{2n+1}$ possible results maximally in measuring AuxBit1, and $|11\dots 11>$ is only one of them. Precise measurement of so numerous qubits is technically difficult and may introduce non-ignorable readout error^34^.

As the only state we focus on is $|11\dots 11>$ in measuring AuxBit1, the other results just offer some redundant information. Thus, we could design a quantum circuit which compresses $|11\dots 11>$ to $|1>$ and the other states to $|0>$.

The step 2 is to propagate the probability of getting $|11\dots 11>$ in measuring AuxBit1 to the probability of getting $|1>$ in another single qubit measurement. This operation can be achieved by using the Control-Not gate that takes multi qubits as its control condition, which reverses the target qubit only if all control qubits are in $|1>$. Construction of such multi-qubits controlled operation using general quantum gate set is introduced in^35^. Generally, defining a controlled operation ${C}^{a}(U)$ acting on $\mathrm{a}+1$ qubits, it can be written as:

3 $${C}^{a}\left(U\right)|{x}_{1}{x}_{2}{x}_{3}\dots {x}_{a-1}{x}_{a}{x}_{a+1}> = |{x}_{1}{x}_{2}{x}_{3}\dots {x}_{a-1}{x}_{a}>{U}^{{x}_{1}{x}_{2}{x}_{3}\dots {x}_{a-1}{x}_{a}}|{x}_{a+1}>$$

2b, where auxiliary qubits 3 (AuxBits3) have a length of 2n and auxiliary qubit 2 (AuxBit2) is the target single qubit we finally wanted.

After obtaining the probability of getting $|1>$ in measuring Auxbit2, we can get the similarity between two binary images by multiplying this probability with ${2}^{2n}$. The factor of ${2}^{2n}$ comes from operations on quantum superposition states. Expanding the comparison to the whole Hilbert space means we compare each of the bases in img-qubits1 with all the bases in img-qubits2, which results in the numerator being the number of identical pixels, but the denominator being square of the number of total pixels.

### Grey images

#### **Algorithm 2**

Compare the similarity between two grey quantum images with a grey range of ${2}^{q}$.

Step 1: Compare the former b pixel value qubits and all pixel position qubits of img-qubits1 and img-qubits2 one by one, where b is an integer less than q, and generate auxiliary qubits 4 (AuxBit4);
Step 2: Compress all-1 qubit strings $|11\dots 11>$ of AuxBit4 into $|1>$ of a single qubit (AuxBit5);
Step 3: Measure AuxBit5 and calculate the similarity s with $\mathrm{s}=\mathrm{p}\times {2}^{2n}$, where p is the probability of getting $|1>$ and ${2}^{2n}$ is the size of images.

Grey images have a much wider application than binary images in classical image processing. In this part, an algorithm for comparing the similarity between two grey quantum images is given. The algorithm is similar to the processing of binary images, except that pixel value qubits compared in step 1 need to be selected.

**Figure 3:**
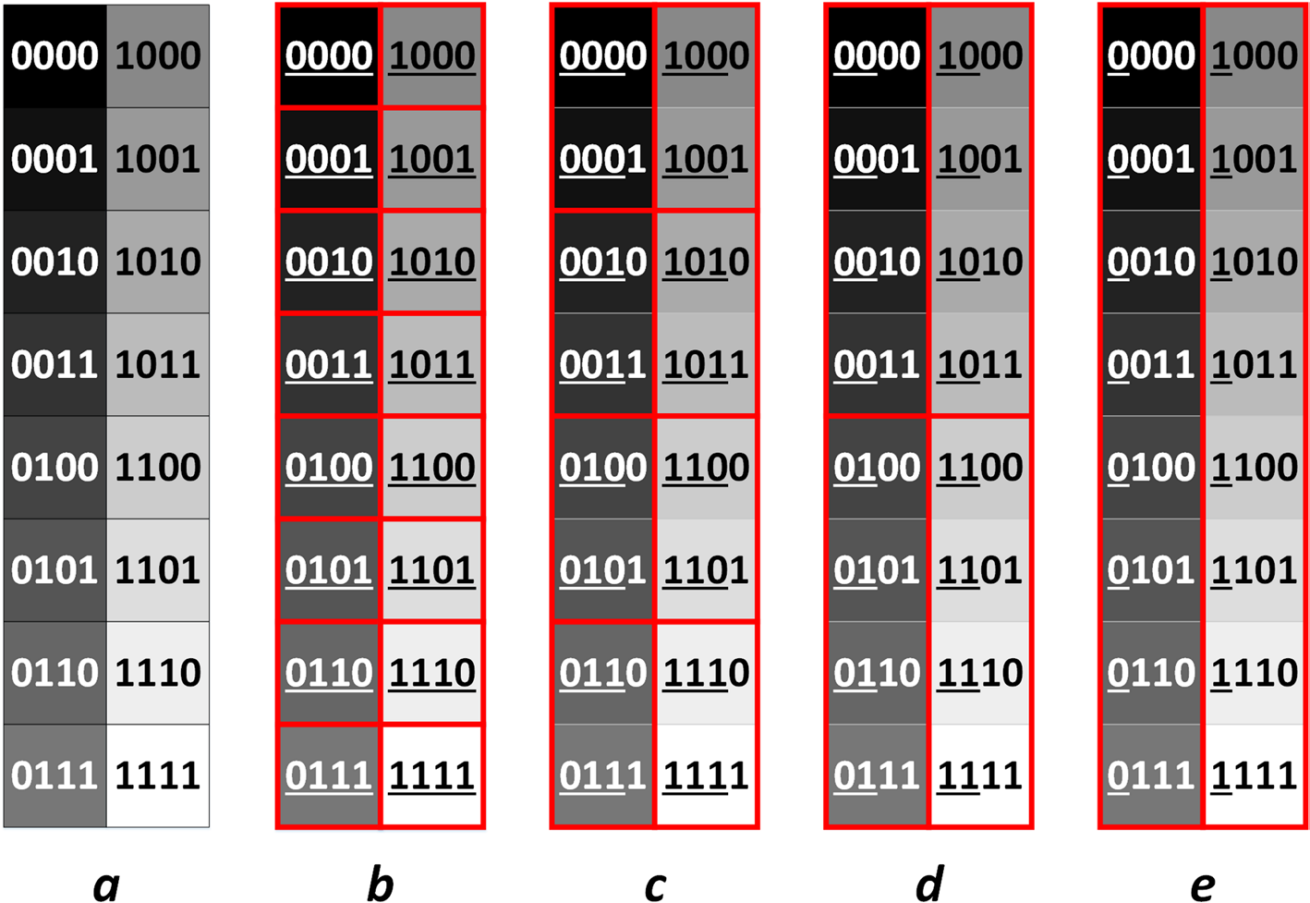
(**a**) The 16 possible states of pixel value qubits under the condition of q = 4. (**b**–**e**) Selecting the former 4,3,2,1 qubits for comparison. The qubit strings in the same red rectangle are considered identical.

For illustration, we will take $\mathrm{q}=4$ at first, and then move on to a more general case. After preparing two grey images in a quantum computer, the quantum state for representing these two images could be written as:

4 $$\frac{1}{{2}^{2n}}\sum_{{Y}_{1}{Y}_{2}=0}^{{2}^{n}-1}\sum_{{X}_{1}{X}_{2}=0}^{{2}^{n}-1}\begin{array}{c}q-1\\ \otimes \\ i=0\end{array}|{C}_{{Y}_{1}{X}_{1}}^{i}{C}_{{Y}_{2}{X}_{2}}^{i}>|{Y}_{1}{X}_{1}{Y}_{2}{X}_{2}>$$

where $|{C}_{{Y}_{1}{X}_{1}}>$ and $|{C}_{{Y}_{2}{X}_{2}}>$ has 16 possible states respectively for $\mathrm{q}=4$, as shown in Fig. 3a.

A vital point for comparing the similarity between grey quantum images is to select proper pixel value qubits of img-qubits1 and img-qubits2 for comparison. Nevertheless, there may exist some drawbacks if inappropriate qubits are selected. For instance, if we compare all qubits of img-qubits1 and img-qubits2, pixels have no contribution to the similarity even if they are only slightly different; if we compare the second and the forth pixel value qubits, states like $|0000>$ and $|1010>$ are considered identical even if their grey values vary greatly.

A qubits selection strategy is presented to avoid these drawbacks. The strategy is to select the former b qubits of $|{C}_{{Y}_{1}{X}_{1}}>$ and $|{C}_{{Y}_{2}{X}_{2}}>$ for comparison. As shown in Fig. 3b–e, if the former 4, 3, 2, 1 qubits are compared, pixels are considered identical if they are in the same red rectangle. For a smaller b, each red rectangle contains more similar pixels. The qubits used for comparison are underlined for clarity.

As for the common grey quantum images having a grey range of ${2}^{q}$, the selection of the former b pixel value qubits indicates a preset of the threshold of ${2}^{q-b}$. From 0 to ${2}^{q}$, every ${2}^{q-b}$ pixel values are considered identical. That is to say, the ${2}^{q}$ grey range is divided into ${2}^{b}$ groups, and all pixel values in the range of $[\mathrm{k}{2}^{q-b},\left(k+1\right){2}^{q-b}-1$], where $\mathrm{k}=0, 1, 2\dots {2}^{b}-1$, are considered identical in comparison.

After step 1, the AuxBit4 was generated based on the quantum state in (4). AuxBit4 has an all-1 qubit string only if the pixel positions are the same and the pixel values are in the same group. Based on the similar analysis in supplement information, the probability of getting the all-1 qubit string in measuring AuxBit4 is equal to $\frac{s}{{2}^{2n}}$, where s is the similarity between two grey images. After step 2, we propagate this intermediate result to the probability of getting $|1>$ in measuring AuxBit5, and the similarity can be calculated by multiplying this probability with ${2}^{2n}$.

### Color images

#### **Algorithm 3**

Compare the similarity between two color quantum images with the RGB component range of ${2}^{q}$.

Step 1: Compare the former ${b}_{R}$, ${b}_{G}$, ${b}_{B}$ (${b}_{R}$, ${b}_{G}$, ${b}_{B}$ are integers less than q) RGB component qubits and all pixel position qubits of img-qubits1 and img-qubits2 one by one, and generate auxiliary qubits 6 (AuxBit6);
Step 2: Compress all-1 qubit strings $|11\dots 11>$ of AuxBit6 into $|1>$ of a single qubit (AuxBit7);
Step 3: Measure AuxBit7 and calculate the similarity s with $\mathrm{s}=\mathrm{p}\times {2}^{2n}$, where p is the probability of getting $|1>$ and ${2}^{2n}$ is the size of images.

Color images could convey more information and are much more common in our daily life than binary and grey images. In this subsection, an algorithm for comparing the similarity between two color images is given. The algorithm is similar to Algorithm 2, except that instead of selecting the former b pixel value qubits of $|{C}_{{Y}_{1}{X}_{1}}>$ and $|{C}_{{Y}_{2}{X}_{2}}>$, we proposed to select the former ${b}_{R}$, ${b}_{G}$, ${b}_{B}$ qubits of $|{R}_{{Y}_{1}{X}_{1}}>$, $|{G}_{{Y}_{1}{X}_{1}}>$, $|{B}_{{Y}_{1}{X}_{1}}>$ and $|{R}_{{Y}_{2}{X}_{2}}>$, $|{G}_{{Y}_{2}{X}_{2}}>$, $|{B}_{{Y}_{2}{X}_{2}}>$ for comparison.

After preparing two color images in a quantum computer, the quantum state for representing these two images could be written as:

5 $$\frac{1}{{2}^{2n}}\sum_{{Y}_{1}{Y}_{2}=0}^{{2}^{n}-1}\sum_{{X}_{1}{X}_{2}=0}^{{2}^{n}-1}\begin{array}{c}q-1\\ \otimes \\ i=0\end{array}|{R}_{{Y}_{1}{X}_{1}}^{i}{G}_{{Y}_{1}{X}_{1}}^{i}{B}_{{Y}_{1}{X}_{1}}^{i}{R}_{{Y}_{2}{X}_{2}}^{i}{G}_{{Y}_{2}{X}_{2}}^{i}{B}_{{Y}_{2}{X}_{2}}^{i}>|{Y}_{1}{X}_{1}{Y}_{2}{X}_{2}>$$

$|{R}_{{Y}_{1}{X}_{1}}{G}_{{Y}_{1}{X}_{1}}{B}_{{Y}_{1}{X}_{1}}{R}_{{Y}_{2}{X}_{2}}{G}_{{Y}_{2}{X}_{2}}{B}_{{Y}_{2}{X}_{2}}>$ has a length of 6q and $|{Y}_{1}{X}_{1}{Y}_{2}{X}_{2}>$ has a length of 4n.

Based on the similar analysis in “Grey images” section, as for the color image having the RGB component range of ${2}^{q}$, the selection of the former ${b}_{R}$, ${b}_{G}$, ${b}_{B}$ RGB component qubits for comparison indicates a preset of the threshold of ${2}^{q-{b}_{R}}$, ${2}^{q-{b}_{G}}$, ${2}^{q-{b}_{B}}$. By setting these thresholds, the red, green, blue ranges are divided into ${2}^{{b}_{R}}$, ${2}^{{b}_{G}}$, ${2}^{{b}_{B}}$ groups, and all RGB component qubits in the range of $[{k}_{R}{2}^{q-{b}_{R}},\left({k}_{R}+1\right){2}^{q-{b}_{R}}-1$], $[{k}_{G}{2}^{q-{b}_{G}},\left({k}_{G}+1\right){2}^{q-{b}_{G}}-1$], $[{k}_{B}{2}^{q-{b}_{B}},\left({k}_{B}+1\right){2}^{q-{b}_{B}}-1$], where ${k}_{R}=0, 1, 2\dots {2}^{{b}_{R}}-1$, ${k}_{G}=0, 1, 2\dots {2}^{{b}_{G}}-1$, ${k}_{B}=0, 1, 2\dots {2}^{{b}_{B}}-1$, are considered identical in comparison.

After step 1, the AuxBit6 was generated based on the quantum state in (5). AuxBit6 has an all-1 qubit string only if the pixel positions are the same and the RGB component values are in the same group respectively. Based on the similar analysis in supplement information, the probability of getting the all-1 qubit string in measuring AuxBit6 is equal to $\frac{s}{{2}^{2n}}$, where s is the similarity between two color images. After step 2, we propagate this intermediate result to the probability of getting $|1>$ in measuring AuxBit7, and the similarity can be calculated by multiplying this probability with ${2}^{2n}$.

## Algorithm complexity

The following discussion of complexity is based on two ${2}^{n}\times {2}^{n}$ binary images, two ${2}^{n}\times {2}^{n}$ grey images with grey range of ${2}^{q}$, and two ${2}^{n}\times {2}^{n}$ color images with RGB component range of ${2}^{q}$. Intuitively, the classical way to calculate the number of identical pixels is to compare every pixel at the same position. As the comparison must be taken one pixel by one pixel, it would take no more than $\mathrm{O}({2}^{2n})$ complexity to complete the calculation for two classical images.

The complexity of a quantum algorithm is usually defined as the number of universal gates used to accomplish the function^20,36^. As shown in Fig. 2a, comparing two single qubits in step 1 costs two CNOT gates and one X gate. Since the comparison is acted on $2+4\mathrm{n}$ qubits for Algorithm 1, the number of basic gates costed in step 1 is no more than $3\times \frac{(2+4\mathrm{n})}{2}=3+6\mathrm{n}$. As shown in Fig. 2b, step 2 needs $2\mathrm{n}$ Toffoli gates and one CNOT gate. Since Toffoli gate can be constructed with six CNOT gates and ten single gates^35^, the number of basic gates costed in step 2 is no more than $1+32\mathrm{n}$. Thus, we need $\left(3+6\mathrm{n}\right)+\left(1+32\mathrm{n}\right)=4+38\mathrm{n}$ gates maximally to complete Algorithm 1. Taking into account the complexity of two binary images preparation^20^, the complexity of comparing the similarity between two binary quantum images is $\mathrm{O}\left({2n2}^{2n}\right)+\mathrm{O}(38\mathrm{n})$.

For Algorithm 2, the comparison is acted on $2\mathrm{b}+4\mathrm{n}$ qubits in step 1. Therefore, the number of basic gates costed in step 1 is no more than $3\mathrm{b}+6\mathrm{n}$, and the number of basic gates costed in step 2 is no more than $16\times \left(\mathrm{b}+2\mathrm{n}-1\right)+1=16\mathrm{b}+32\mathrm{n}-15$. Thus, we need $\left(3\mathrm{b}+6\mathrm{n}\right)+\left(16\mathrm{b}+32\mathrm{n}-15\right)=19\mathrm{b}+38\mathrm{n}-15$ gates maximally to complete Algorithm 2. Taking into account the complexity of two grey images preparation^20^, the complexity of comparing the similarity between two grey quantum images is $\mathrm{O}\left({2qn2}^{2n}\right)+\mathrm{O}(19\mathrm{b}+38\mathrm{n})$.

For Algorithm 3, the comparison is acted on ${2b}_{R}+{2b}_{G}+{2b}_{B}+4\mathrm{n}$ qubits in step 1. Therefore, the number of basic gates costed in step 1 is no more than $3{b}_{R}+3{b}_{G}+3{b}_{B}+6\mathrm{n}$, and the number of basic gates costed in step 2 is no more than $16{b}_{R}+{16b}_{G}+{16b}_{B}+32\mathrm{n}-15$. Thus, we need ($3{b}_{R}+3{b}_{G}+3{b}_{B}+6\mathrm{n})+\left(16{b}_{R}+{16b}_{G}+{16b}_{B}+32\mathrm{n}-15\right)=19{b}_{R}+{19b}_{G}+{19b}_{B}+38\mathrm{n}-15$ gates maximally to complete Algorithm 3. Taking into account the complexity of two color images preparation^17^, the complexity of comparing the similarity between two color quantum images is $\mathrm{O}\left(6\mathrm{q}+4\mathrm{n}+12\mathrm{qn}{2}^{2n}\right)+\mathrm{O}(19{b}_{R}+{19b}_{G}+{19b}_{B}+38\mathrm{n})$.

The time complexity of previous quantum algorithms that compare the similarity between two quantum images based on NEQR or NCQI is given in Table 2. In order to reflect the advantage of the proposed algorithms, the data in Table 2 don’t contain the complexity of image preparation as this process is constant in almost all QImP algorithms. The complexity of SAB_PV, SAG_SAB_PV, SAC_SAB_PV depends on the expectation of the precision of results and the data in Table 2 are from an instance given in^20^. In addition, because $b$, ${b}_{R}$, ${b}_{G}$, and ${b}_{B}$ are integers less than q, the complexity of Algorithms 2 and 3 in Table 2 reflects the maximum complexity taking ${b,b}_{R},{b}_{G},{b}_{B}=q$. Even in this worst case (all pixel value qubits are compared), an exponential acceleration is still achieved than the previous algorithms.

**Table 2:** Complexity of different quantum methods for comparing the similarity between two quantum images based on NEQR and its variant.

Algorithms	Image style	Complexity
Algorithm 1	Binary	$\mathrm{O}(38\mathrm{n})$
Algorithm 2	Grey	$\mathrm{O}(19\mathrm{q}+38\mathrm{n})$
Algorithm 3	Color	$\mathrm{O}(57\mathrm{q}+34\mathrm{n})$
Classical method	Binary/grey/color	$\mathrm{O}({2}^{2n})$
SAB_PV^20^ ($t=n+5$)	Binary	$\mathrm{O}\left(64\mathrm{n}{2}^{3n}\right)$
SAG_SAB_PV^20^ ($t=n+5$)	Grey	$\mathrm{O}\left(2\mathrm{qn}{2}^{2n}\right)+\mathrm{O}\left(48{q}^{2}\right)+\mathrm{O}\left(64\mathrm{n}{2}^{3n}\right)$
SAC_SAB_PV^20^ ($t=n+5$)	Color	$\mathrm{O}\left(12\mathrm{qn}{2}^{2n}\right)+\mathrm{O}\left(6{q}^{3}\right)+\mathrm{O}\left(64\mathrm{n}{2}^{3n}\right)$
Algorithm in^37^	Binary/grey	$\mathrm{O}(28\mathrm{n}{2}^{2n})$

The complexity of image preparation is not contained in this table.

## Calculation results

In the following, some calculation results of binary, grey, and color images are given based on either real quantum computer or simulations.

### Experiment result of binary images

The best method for validating a quantum algorithm is to run it on a real quantum computer. IBMQ Experience offers several real quantum computers which can be accessed by public via cloud, and there have been demonstrations of various quantum algorithms based on these quantum computers^38–40^. The similarity between three binary images is calculated using IBM_Manila. IBM_Manila is a real quantum computer with five qubits and 32 quantum volume^41^. The average CNOT gate error of IBM_Manila is 0.8106% and the average single qubit readout error of IBM_Manila is 2.102% (updated aperiodically)^42^.

**Figure 4:**
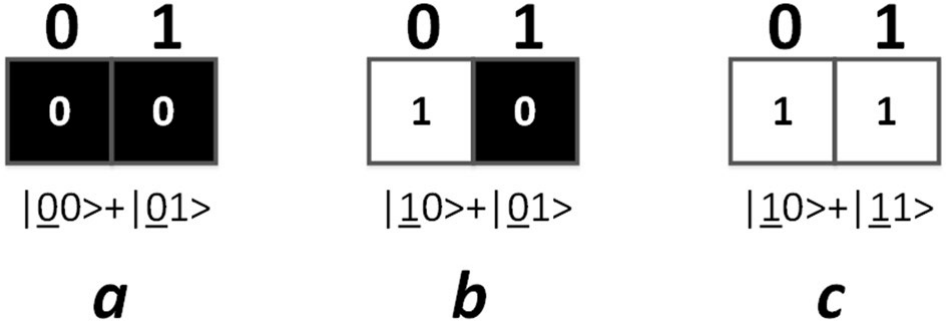
Three binary images used for calculating similarity. The image representation states are shown below the images. The pixel value qubits are underlined. Probability amplitudes are not given for simplification.

Due to the limited qubits of IBM_Manila, we have to make some simplifications in order to apply Algorithm 1. The simplifications include: the binary images for comparison have only 2 pixels, a pixel value qubit is reused as part of Auxbit1, and the Auxbit1 is measured directly for calculating the similarity. The binary images used for experiments are shown in Fig. 4.

**Figure 5:**
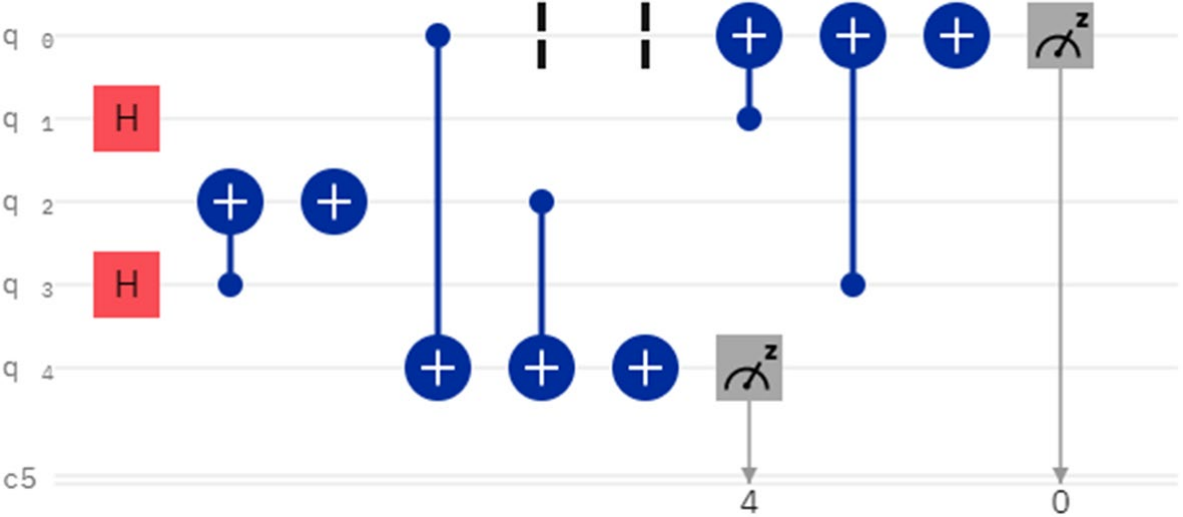
Quantum circuit for comparing similarity between image a and b in Fig. 4.

A quantum circuit example is given in Fig. 5. Q0 and q1 are used for representing image a. Q2 and q3 are used for representing image b. After images were prepared, q4 is used for comparing pixel value qubits of image a and b. Due to the lack of more qubits, q0 is reused for comparing pixel position qubits of image a and b. For the same reason, q0 and q4 are measured directly for calculating the similarity between image a and b.

For IBM_Manila, the number of runs of circuits can be selected from 1 to 8192. In more runs, we can get results closer to expectations. The circuit in Fig. 5 was run 8192 times on IBM_Manila, and the numbers of getting $|11>$ in measuring q0 and q4 were counted. In an instance, the numbers of getting $|11>$ were 1810. Thus, the similarity between image a and b was calculated as $\frac{1810}{8192}\times 2=44.19\%$. The similarity between image a and c, image b and c was calculated similarly, and the results were 8.40% and 46.44%. There is a notable margin between the calculation results and expectations. This is mainly because of the readout error and imperfect quantum gates. In addition, the limitation of physical links between qubits on a real quantum chip also introduces additional SWAP gates when compiling quantum circuits^43^.

### Simulation result of grey images

On account of the limited qubits and fidelities, it is unlikely to run a complex quantum circuit on the real quantum computer and obtain a satisfied result presently. IBMQ Experience also offers several simulators that have been frequently used for demonstration of quantum algorithms^44–46^. IBMQ_qasm_simulator is a general-purpose simulator for simulating quantum circuits both ideally and subject to noise modeling^42^.

**Figure 6:**
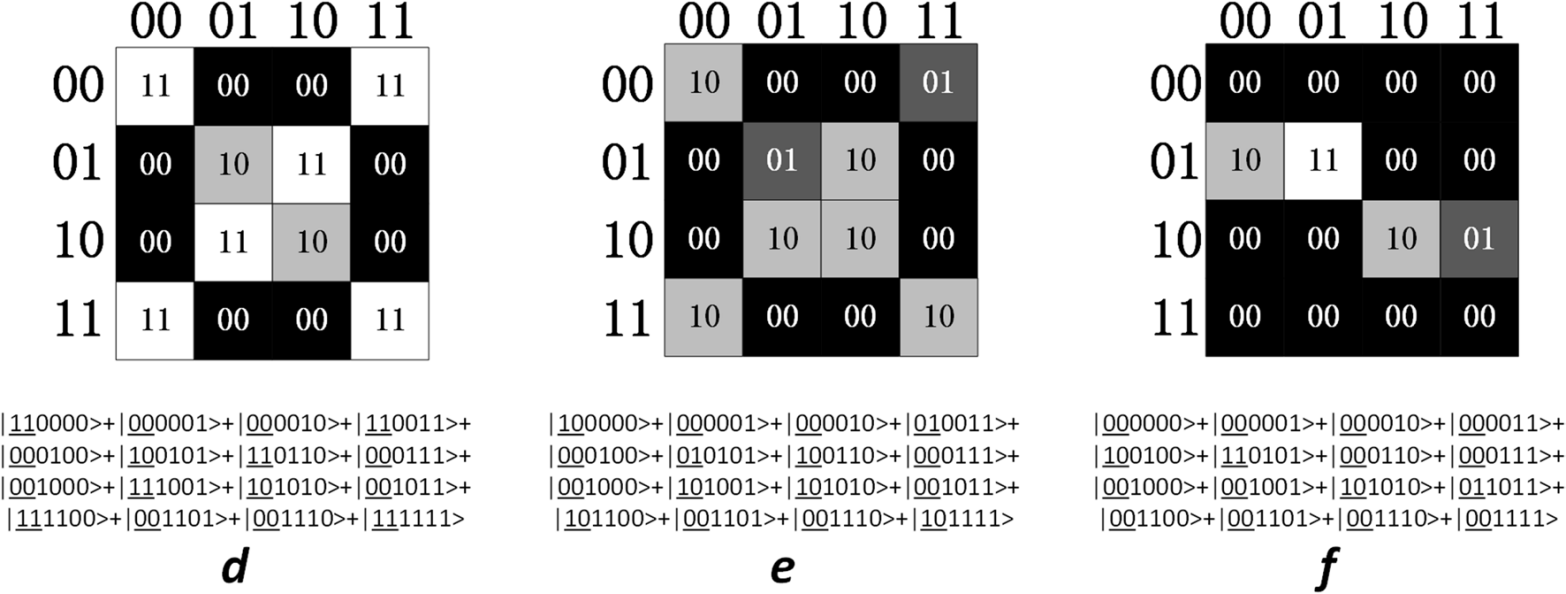
Three grey images used for calculating similarities. The image representation states are shown below the images. The pixel value qubits are underlined. Probability amplitudes are not given for simplification.

**Figure 7:**
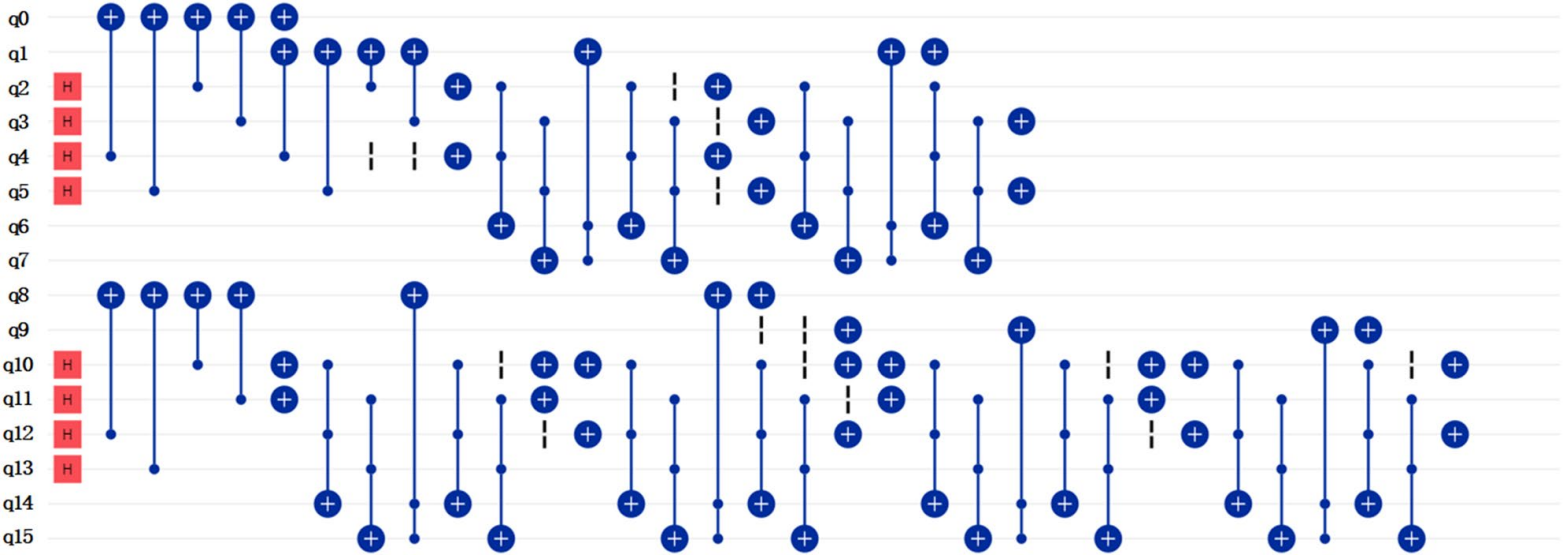
Quantum circuit for preparing image d and e in Fig. 6. Q0 to q5 are qubits representing image d. Q8 to q13 are qubits representing image e. Other qubits are ancillary qubits used for helping preparation. The other part of the algorithm is omitted for clarity.

Similarities between three grey quantum images (Fig. 6) are calculated using IBMQ_qasm_simulator. The example of preparing image d and e is given in Fig. 7. The other part of the algorithm can be generated with reference to Fig. 2 and is omitted in Fig. 7 for clarity. For IBMQ_qasm_simulator, the number of runs of circuits can be selected from 1 to 8192. In order to get results closer to expectations, the quantum circuits were run 8192 times. The similarity results are shown in Table 3. If all pixel value qubits are compared, there is not so significant difference between the three similarity results. By selecting the first pixel value qubit for comparison, we can get an obvious maximum similarity when comparing image d and e, which is consistent with expectation as they both look like number “0” but image f looks like number “2”. Other similarity results also have an increment by transforming the comparison on all pixel value qubits to on the first pixel value qubit.

**Table 3:** Similarities between three sample grey images.

**All pixel value qubits are compared**	
Similarity between image d and e	53.13%
Similarity between image e and f	40.23%
Similarity between image d and f	41.02%
**The first pixel value qubit is compared**	
Similarity between image d and e	86.13%
Similarity between image e and f	54.30%
Similarity between image d and f	54.69%

### Simulation result of color images

**Figure 8:**
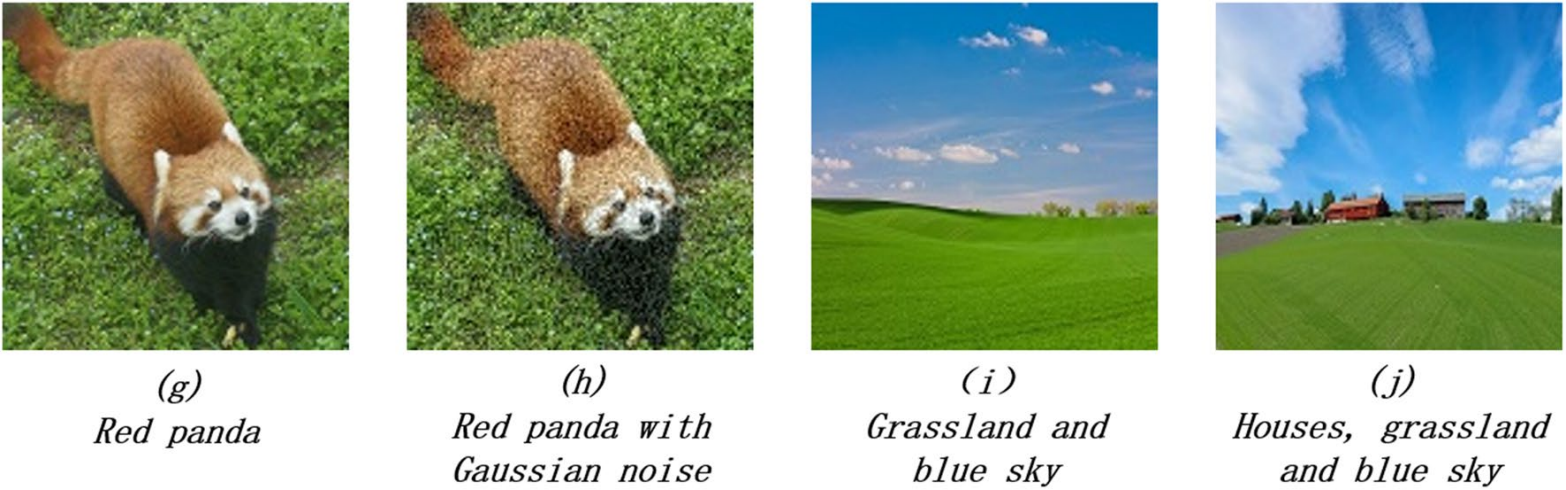
Four color images used for calculating similarities.

Color images used for experiments are 8-bits per RGB channel and composed of $128\times 128$ pixels (Fig. 8). Thus, it needs $\left(8\times 3+7\times 2\right)\times 2=76$ qubits for representing two images. Even if only Auxbit6 is included, the Algorithm 3 needs 114 qubits at least to be simulated. Such quantum system has exceeded the capability of gate-based simulators.

In order to simulate the evolution of quantum states in the algorithm, some simplifications have to be made from Algorithm 3. Firstly, in step1, instead of multiplying the bases with unitary matrices which have the enormous dimensions, the Auxbit6 was generated directly according to its truth table (Table 1). Secondly, the number of Auxbit6 being $|11\dots 11>$ was counted for statistical analysis of the expected probability of getting $|11\dots 11>$ in measuring Auxbit6. Finally, instead of step3, this probability was used for calculating the similarities. Similar simplifications are also used in^20^.

The simulation was carried out on a laptop with Core i7-8565U CPU and 16 GB RAM. The pixel values and positions of sample images were extracted using the open-source software OpenCv. The subsequent calculations were based on the array computing package NumPy with python. The calculation results for three different selections of ${b}_{R}$, ${b}_{G}$, and ${b}_{B}$ are given in Table 4. In all cases, image g and h have the largest similarity, which is consistent with prediction as they look the most similar visually. Image i and j also have a similarity more than 50% when ${b}_{R}$, ${b}_{G}$, ${b}_{B}=1$. This similarity has a rapid decrease when ${b}_{B}$ is increased to 2. However, it doesn’t fall so quickly when ${b}_{G}$ is increased to 2. We suppose that this is due to the large proportion of blue part in image i and j resulting in the great impact of blue components on similarity.

**Table 4:** Similarities between Four color images.

${{\varvec{b}}}_{{\varvec{R}}}=1$, ${{\varvec{b}}}_{{\varvec{G}}}=1$, ${{\varvec{b}}}_{{\varvec{B}}}=1$					
g and h	g and i	g and j	h and i	h and j	i and j
85.27%	14.90%	16.58%	14.73%	16.37%	53.42%
${{\varvec{b}}}_{{\varvec{R}}}=1$, ${{\varvec{b}}}_{{\varvec{G}}}=2$, ${{\varvec{b}}}_{{\varvec{B}}}=1$					
g and h	g and i	g and j	h and i	h and j	i and j
81.60%	12.65%	13.57%	12.37%	13.42%	49.09%
${{\varvec{b}}}_{{\varvec{R}}}=1$, ${{\varvec{b}}}_{{\varvec{G}}}=1$, ${{\varvec{b}}}_{{\varvec{B}}}=2$					
g and h	g and i	g and j	h and i	h and j	i and j
78.87%	10.49%	11.40%	10.23%	10.98%	30.87%

## Conclusion

In this study, three novel algorithms are proposed to compare the similarity between two quantum images. The algorithms are suitable for binary, grey, and color images respectively. Compared with existing methods, especially with the algorithms also based on NEQR and NCQI^20,37^, our work has the following contributions.

Firstly, under the condition that the image preparation is not included, the proposed algorithms can achieve exponential acceleration than the previous and classical methods.

Secondly, the binarization of grey and color images in^20^ is slightly deficient due to the lack of available quantum circuits. Our study introduces a novel method for comparing the similarity between two binary, grey or color images, which not only benefits from extra acceleration, but also makes a progress in assessing the similarity of grey and color images based on NEQR and NCQI.

Finally, although it is a primitive demonstration with binary images that have only two pixels, this is the first time to compare the similarity between two quantum images on a real quantum computer to the best of our knowledge.

This study is a preliminary attempt of the practical application of quantum computing in the image processing field. Experiment and simulation results have indicated the effectiveness of the algorithms. However, circuit depth and connectivity limit the application of our algorithms on a near-term quantum computer. As shown in Table 4, an inappropriate selection of pixel value qubits in comparing color images may also cause a significant decrease of results. A qubits selection strategy with a circuit optimization remains to be developed for future studies.

## Supplementary Information

Supplementary Information.
