# Supplementary material for: Comparison of the similarity between two quantum images
Source: Sci Rep. 2022 May 11;12:7776. doi: 10.1038/s41598-022-11863-9 (PMC9095865; doi:10.1038/s41598-022-11863-9)
Supplement: Supplementary file 1 — Supplementary Information. [file 41598_2022_11863_MOESM1_ESM.pdf]

Supplementary Information for "Compare the Similarity between Two Quantum Images"

You-hang Liu, Zai-dong Qi, Qiang Liu

After preparing two binary images in a quantum computer based on NEQR, the quantum state for representing these two images could be written as:

$$\frac{1}{2^{2n}}\sum_{Y_1Y_2=0}^{2^n-1}\sum_{X_1X_2=0}^{2^n-1}|C_{Y_1X_1}^0C_{Y_2X_2}^0>|Y_1X_1Y_2X_2> \qquad \text{(S1)}$$

where $|Y_1X_1Y_2X_2>$ represents pixel positions and $|C_{Y_1X_1}^0C_{Y_2X_2}^0>$ represents pixel values that have only four possible states, $|00>$, $|01>$, $|10>$, or $|11>$.

The first step in algorithm 1 is to compare all qubits in img-qubits1 and img-qubits2 one by one. After step 1, the AuxBit1 (composed of $|A_C>$ and $|A_{Y_1X_1Y_2X_2}>$) is generated based on the quantum state in (S1) and Table 1:

$$\frac{1}{2^{2n}}\sum_{Y_1Y_2=0}^{2^n-1}\sum_{X_1X_2=0}^{2^n-1}|C_{Y_1X_1}^0C_{Y_2X_2}^0>|A_C>|Y_1X_1Y_2X_2>|A_{Y_1X_1Y_2X_2}> \qquad \text{(S2)}$$

where the length of $|A_C>$ is 1 and the length of $|A_{Y_1X_1Y_2X_2}>$ is 2n. $|A_C>$ has a state of $|1>$ only if $|C_{Y_1X_1}^0C_{Y_2X_2}^0>$ has a state of $|00>$ or $|11>$, which indicates the identical pixel values. $|A_{Y_1X_1Y_2X_2}>$ has a state of all-1 qubit string $|1\ldots11>$ only if $Y_1=Y_2$ and $X_1=X_2$, which indicates the identical pixel positions. Therefore, all-1 qubit strings $|11\ldots11>$ (the following is written as $|11\ldots11>$ for simplification) of $|A_CA_{Y_1X_1Y_2X_2}>$ indicate the identical pixel positions and values.

Supposing two quantum images composed of $2^n\times2^n$ pixels have j (j is an integer less than 2^{2n}) identical pixels, there are j $|11\ldots11>$ strings of $|A_CA_{Y_1X_1Y_2X_2}>$ in (S2), and the similarity equals $\frac{j}{2^{2n}}$, where 2^{2n} is the number of the total pixels.

Focusing on the subspace of AuxBit1, in measuring quantum state in (S2), the probability of getting each basis is $\frac{1}{2^{4n}}$. As there are j $|11\ldots11>$ strings of $|A_CA_{Y_1X_1Y_2X_2}>$, the probability of getting $|11\ldots11>$ equals $\frac{j}{2^{4n}}$. That is to say, after measuring AuxBit1, we can calculate the similarity between two quantum images by multiplying the probability of getting $|11\ldots11>$ with 2^{2n}.
